# Endoscopic transpapillary drainage in disconnected pancreatic duct syndrome after acute pancreatitis and trauma: long-term outcomes in 31 patients

**DOI:** 10.1186/s12876-019-0977-1

**Published:** 2019-04-16

**Authors:** Yan Chen, Yueping Jiang, Wei Qian, Qihong Yu, Yuanhang Dong, Huiyun Zhu, Feng Liu, Yiqi Du, Dong Wang, Zhaoshen Li

**Affiliations:** 1 Department of Gastroenterology, Digestive Endoscopy Center, Changhai Hospital, The Second Military Medical University, 168 Changhai Road, Shanghai, 200433 China; 2 Digestive Endoscopy Center, Changhai Hospital, The Second Military Medical University, Shanghai, China; 3 Department of Gastroenterology, Affiliated Hospital of Qingdao University, Shandong, China; 4 Department of Gastroenterology, Center of Clinical Epidemiology and Evidence-Based Medicine, The Second Military Medical University, Shanghai, China

**Keywords:** Pancreatic fistula, Endoscopic retrograde cholangiopancreatography, Acute abdominal trauma, Acute necrotizing pancreatitis

## Abstract

**Background:**

Conventionally, disconnected pancreatic duct syndrome is treated surgically. Endoscopic management is associated with lesser morbidity and mortality than that observed with surgery and shows similar success rates. However, limited data are available in this context. We evaluated the efficacy of endotherapeutic management for this syndrome.

**Methods:**

We prospectively obtained data of patients with disconnected pancreatic duct syndrome between September 2008 and January 2016. Demographic and clinical data were assessed, and factors affecting clinical outcomes were statistically analyzed.

**Results:**

Thirty-one patients underwent 40 endoscopic transpapillary procedures, and 1 patient developed an infection after prosthesis insertion. Etiological contributors to disconnected pancreatic duct syndrome were abdominal trauma (52%) and acute necrotizing pancreatitis (48%). The median interval between the appearance of pancreatic leaks and disconnected pancreatic duct syndrome was 6.6 months (range 0.5–84 months). The median follow-up after the last treatment procedure was 38 months (range 17–99 months). Patients with complete main pancreatic duct disruption in the body/tail showed a low risk of pancreatic atrophy (*P* = 0.009). This study highlighted the significant correlation between endoscopic transpapillary drainage and clinical success (*P* = 0.014).

**Conclusions:**

Disconnected pancreatic duct syndrome is not an uncommon sequel of pancreatic injury, and much of the delayed diagnosis is attributable to a lack of knowledge regarding this disease. Endoscopic transpapillary intervention with ductal stenting is an effective and safe treatment for this condition.

## Background

Disconnected pancreatic duct syndrome (DPDS) most commonly occurs as an adverse effect of acute necrotizing pancreatitis (ANP) or secondary to abdominal trauma [1]. The incidence of DPDS with ANP in our center was less than 2%. Chronic pancreatitis, pancreatic malignancies, and abdominal surgery can also cause DPDS. DPDS causes pancreatic ductal (PD) leakage owing to a complete discontinuity of the main pancreatic duct(MPD) and isolation of the entire upstream pancreatic segment (e.g., body or tail), which does not communicate with the duodenal papilla. Because of persistent pancreatic leak, diagnosis of DPDS is then considered, and clinically DPDS most commonly presents with a nonhealing external pancreatic fistula (EPF) with high amylase activity or peripancreatic fluid collections (PFC) refractory to conservative management. These conditions cause further complications including intra-abdominal sepsis, hemorrhage from peripancreatic blood vessels, and exocrine or endocrine pancreatic insufficiency [2–4]. Thus, early diagnosis and treatment are important, which unfortunately are not possible in clinical practice.

Pancreatography (magnetic resonance cholangiopancreatography [MRCP] or endoscopic retrograde cholangiopancreatography [ERCP]) is the most reliable modality to accurately diagnose DPDS. An abrupt discontinuity in the MPD observed during ERCP is diagnostic of DPDS with or without contrast extravasation [5, 6].

Potential treatment for DPDS includes conservative, endoscopic, and surgical measures or the use of interventional radiology. Primary percutaneous drainage is being replaced by endoscopic drainage, which prevents EPF formation [6]. Although surgical and endoscopic procedures are equally effective in draining non-resolving symptomatic pancreatic pseudocysts, endoscopy is a cost-effective option associated with a shorter hospital stay [7]. Surgical management has demonstrated a mortality rate of 6–9% and a recurrence rate of 23–60% [8–11]. To date, there is no consensus regarding the optimal endoscopic treatment for DPDS. Long-term indwelling transluminal stents are currently recommended, albeit with inadequate supporting evidence because this is a rare condition [5, 12–14]. Endoscopic transpapillary drainage (ETD) was considered safe and effective treatment in cases of partial PD disruption where a bridging endoprosthesis could be used; however, it was shown to be ineffective to treat DPDS, with a low reported success rate of 26–43% [5, 12, 15, 16]. However, most of these studies were performed at a few expert centers and included a small cohort of patients with DPDS without long-term follow-up.

We report 31 consecutive patients with DPDS who underwent a diagnostic and therapeutic ERCP with long-term follow-up. Moreover, we propose that transpapillary stent insertion improves clinical outcomes in patients with DPDS when the site of disruption is not bridged.

## Methods

We performed a retrospective analysis of a prospectively obtained ERCP database. We investigated every patient who developed DPDS after ANP or abdominal trauma between September 2008 and January 2016. Most of them were treated in local hospitals, they came to our center for endoscopic treatment of this delayed complication when the condition of ANP or abdominal trauma was stable. Electronic medical records were reviewed. Variables included patient demographics, clinical manifestations, etiology, therapy performed (operation, percutaneous or transmural drainage), ERCP findings and intervention, other endoscopic management, adverse events, clinical course and outcomes (success, failure, and number of procedures performed). The study protocol was approved by the Changhai Hospital Ethics Committee and it conforms to the provisions of the Declaration of Helsinki. All patients provided written informed consent. Pre-procedure evaluation of all disconnected viable pancreatic tissue was performed using cross-sectional imaging (contrast-enhanced computed tomography [CT] or MRCP). We included patients with ANP or trauma with an ERCP-proven diagnosis of DPDS.

### Definitions

We defined DPDS as total disruption of the MPD such that a guidewire could not traverse this disconnection, with nonopacification of the PD upstream from the site of disruption. Technical success meant successful Proximal PD opacification regardless of PD cannulation. Clinical success and failure were assessed in terms of radiological and clinical response. Clinical success was defined as the absence of symptoms and a continuous decrease in cutaneous leaks or in the size of PFCs after the initial procedure. Clinical failure was defined as persistent or recurrent PFCs or EPFs or no clinical improvement and/or obvious deterioration. Interval of DPDS was the time lapse between the appearance of PFCs/EPF after pancreatic injury and the diagnosis of DPDS. Follow-up time was defined as the time between the last endoscopic therapy or surgery and the last clinical follow-up, although our follow-up commenced with the diagnosis of DPDS. If stent migration was observed in the interval between 2 consecutive follow-up examinations, the duration between stent placement and the time of last imaging was considered the stent-retention time. Pancreatic debridement indicated that the surgical debridement was performed before ERCP.

### Technique

PD cannulation was initially attempted using a floppy tip guidewire. If the duct could not be accessed, a small volume of contrast material was injected via a catheter to identify and define the MPD. We attempted to bridge the disruption in all patients, although this was not possible in all. Once DPDS had been diagnosed, therapeutic endoscopists performed transpapillary intervention. Four options were considered: stent insertion, sphincterotomy, a combination of stent insertion and sphincterotomy, and pancreatography under ERCP. Stent insertion referred to ETD. The choice of stent was based upon specific ductal anatomy. Strictures were dilated in a step-wise manner using 3, 4 and 5F dilators. A 5- or 7-Fr transpapillary pancreatic stent was subsequently advanced over the guidewire into the PD. In several patients, only pancreatography was performed without any treatment based on the concept that the best results following ETD were obtained if endoprosthetic bridging of the MPD disruption was performed. In these patients, a feeding tube was placed for enteral nutrition while awaiting other treatments such as surgery. All procedures were performed at the discretion of the 4 treating endoscopists.

### Post-treatment follow-up

Post-treatment follow-up included ≥1 office visits to assess clinical variables, imaging study results, and post-ERCP outcomes. Serial abdominal CT examinations were performed in patients who underwent transpapillary therapy at 2- to 3-week intervals until resolution was confirmed. Cross-sectional imaging studies were performed before stent removal, which was usually performed 3 months after placement. New stent placement was required in patients with a persistent fistula or radiographically proven persistent PFC. Transpapillary re-intervention was not required in patients with clinical improvement and/or complete resolution of PFCs or EPF. To assess long-term outcomes, follow-up data were obtained via 3-monthly telephonic interviews with patients or relatives, who were questioned specifically regarding any evaluation or treatment required for recurrent PFCs/EPFs, as well as the clinical status and adverse events including chronic pancreatitis following endoscopic treatment. All patients were followed up from the date DPDS was diagnosed until November 2017. Follow-up included a minimum duration of 17 months from the last endoscopic or surgical treatment.

### Statistical analysis

Patients were grouped based on the long-term clinical outcome (successful vs. unsuccessful). Characteristics of patients with PD stent insertion and those without stent insertion were compared using the Student t-test for normally distributed continuous data. The Wilcoxon test was used for abnormally distributed continuous data and the Fisher exact test for categorical data. Possible confounders associated with ETD were also analyzed.

## Results

### Patient demographics

We identified 31 patients with DPDS from 10 different provinces of China (24 men, 77.4%), with a median age of 36 years (range 23–70 years). Regarding the etiology, in 16 patients (51.6%) we observed trauma-induced DPDS—work-related [8], car accident-related [6], assault-related [1], or fall-related [1]. The second most common etiology was acute pancreatitis (15 patients, 48.4%)—caused by gallstones [11], hyperlipidemia [2], and idiopathic causes [2]. Fifteen patients (48.4%) showed PFCs and 16 (51.6%) showed EPFs. All 16 fistulas extended to the skin and occurred after radiological [11] or surgical [5] drain placement. Patients with ANP were more likely to have undergone percutaneous drainage before ERCP than patients with trauma (9/15 vs. 2/16, *P* = 0.006) (Table 1). All patients were symptomatic with abdominal pain or intolerance to oral intake. No clinical infection or gastrointestinal obstruction occurred in these patients with a delayed diagnosis.

**Table 1:** Main characteristics of patients with DPDS

Characterristics	ANP *N* = 15	Trauma *N* = 16	*p* value
Patient characteristics before ERCP			
Etiology of AP			
Biliary	11		
Nonbiliary	4		
Etiology of Trauma			
Work		8	
Car accident		6	
Personal fight		1	
Fall down		1	
Age (year)^a^	42 (25~70)	32 (23~54)	0.051
Male	3 (20%)	4 (27%)	> 0.99
PFCs: EPFs	5:10	10:6	0.104
Diameter of PFC (cm)^a^	5.2 (2.5~8.9)	2.4 (2.0~11.4)	0.268
CT guided puncture before ERCP	9 (60%)	2 (12.5%)	0.006
EUS guided puncture before ERCP	2 (13.3%)	0 (0%)	0.226
Pancreatic debridement before ERCP	2 (13.3%)	7 (43.8%)	0.113
Findings at ERCP and therapy			
Interval between discovering pseudocyst or fistula and DPDS (months) ^a^	3.6 (0.5~38)	7.5 (1.0~84)	0.166
Location			0.704
Proximal (head or neck)	10	12	
Distal (body or tail)	5	4	
Endoscopictherapy			
Transpapillary drainage^b^	8	5 (7)	
Sphincterotomy^b^	0 (1)	3 (4)	
Transpapillary drainage + Sphincterotomy	2	0	
No intervention^b^	3 (4)	5	
Findings at follow-up			
Following up Lost	1 (6.7%)	4 (25%)	0.333
Stent retention (days) ^a^	95 (15~356)		
Follow-up time after Last ERCP intervention or surgery (months)^a^	38 (17~99)		
≥2 stent insertion	3 (20%)	2 (12.5%)	0.654
Successful outcome	10 (71.4%)	8 (66.7%)	> 0.99
Developed DM	2 (14.3%)	1 (8.3%)	> 0.99
Developed diarrhea	3 (21.4%)	0 (0%)	0.225
Developed atrophy	6 (42.9%)	4 (33.3%)	0.701

^a^Described as median with range

^b^Numbers in parentheses indicate patients who have not been followed up

PFCs peripancreatic fluid collections, EPFs external pancreatic fistulas, DM diabetes mellitus

### Characteristics of disconnected pancreatic duct syndrome and route of procedure

**Fig. 1:**
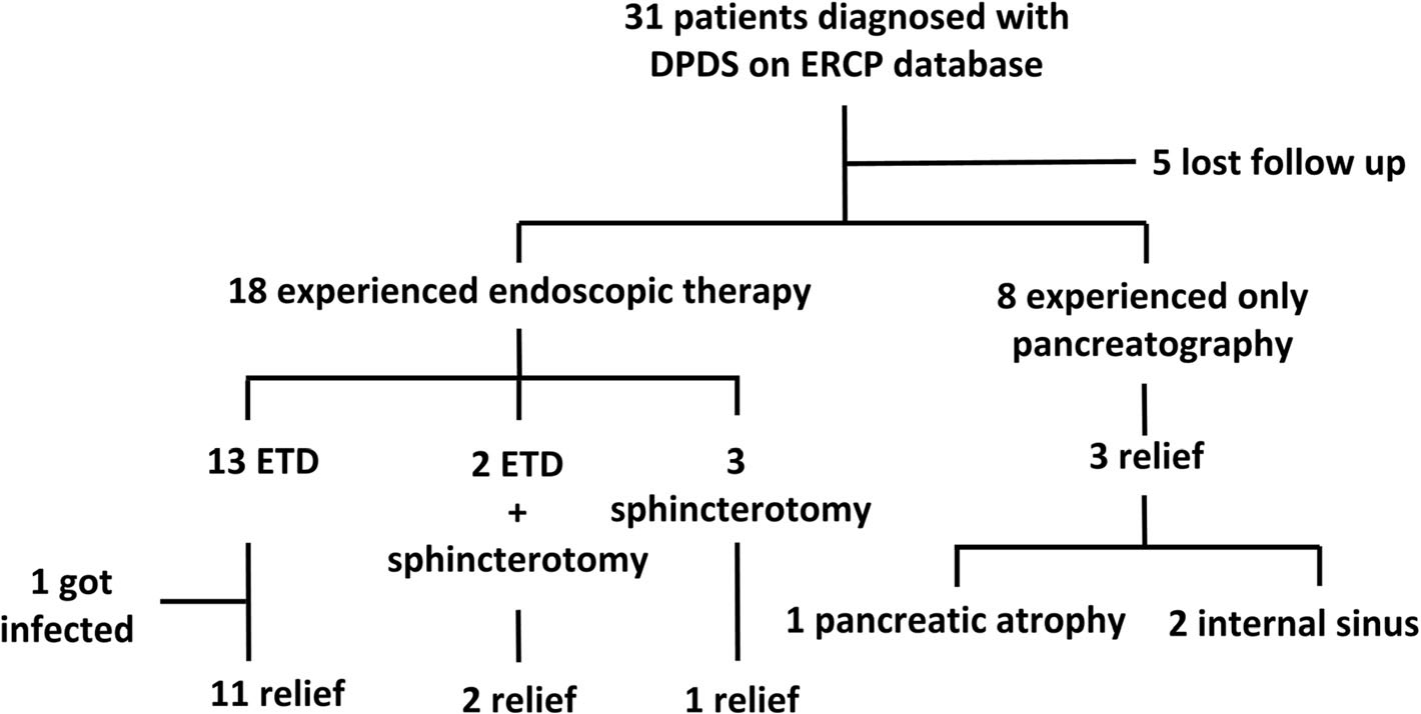
Flowchart of 31 patients. DPDS: disconnected pancreatic duct syndrome; ERCP: endoscopic retrograde cholangiopancreatography; ETD: endoscopic transpapillary drainage

The median diameter of PFCs was 2.5 cm (range 2.0–11.4 cm). Daily leakage of EFCs was approximately 200–400 mL. The median interval between the appearance of pancreatic leaks and the diagnosis of DPDS was 6.6 months (range 0.5–84 months). Delayed diagnosis was secondary to a lack of awareness regarding this disease and the long-term dependence on conservative treatments including medicines and long-term observation. Pancreatography demonstrated complete MPD disruption in the following pancreatic locations: head or neck (22 patients, 71%), and body or tail (9 patients, 29%). The success rate did not statistically significantly differ between proximal and distal PD disconnections. Sphincterotomy alone was used in 5 patients (16.1%), and concomitantly with transpapillary stent insertion in 2 (6.5%). ETD was completed in 17 of 31 (54.8%) patients (Fig. 1). The stent was inserted with its proximal end adjacent to or entering disruption. We used 7-Fr (29.2%) and 5-Fr (71.8%) stents. No significant differences were observed among these mentioned characteristics.

### Adverse events

**Fig. 2:**
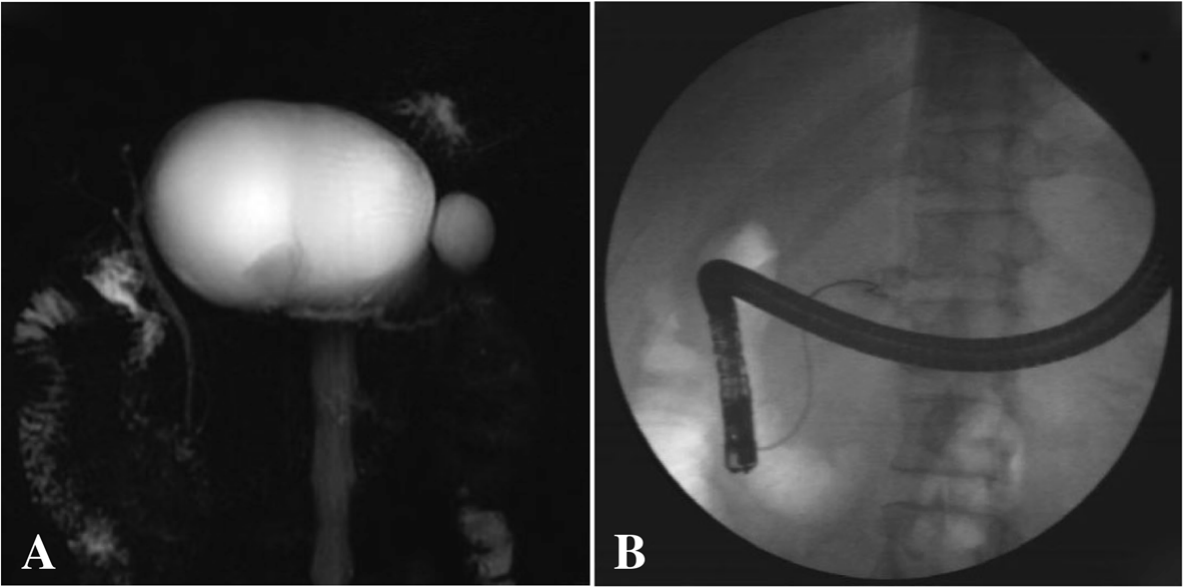
The case of cyst infection after prosthesis drainage under ERCP. **a**, A fluid collection (11.4 cm) with dilated upstream MPD on MRCP; **b**, MPD cannot be bridged with guide wire and extravasation of contrast into pseudocyst, without visualizationof the MPD at the body or tail at ERCP

No mortality occurred in the 31 patients studied, and the technical success rate was 100% with only 1 adverse event (development of an infected PFC [11.4 cm in diameter] in a patient with trauma-induced DPDS, Fig. 2) after prosthesis insertion. This patient required endoscopic ultrasound-guided transmural cystoduodenostomy with placement of a plastic stent and 7 days of hospitalization and recovered 3 months later. We did not consider stent migration among adverse events and attributed this to PD decompression.

### Clinical success

Clinical success was observed in 17 patients. There were no statistically significant intergroup differences regarding clinical success/failure, and ERCP findings except that those who underwent ETD showed higher rates of clinical success than those who needed additional therapy (*P* = 0.014) (Table 2).

**Table 2:** DPDS characteristics according to clinical outcome

Characteristics	Successful outcome *N* = 17	Clinical failure and need surgery *N* = 9	*p* value
Age (year)^a^	36 (23~70)	36 (25~53)	0.817
Male	12 (70.6%)	8 (88.9%)	0.380
Etiology			0.683
ANP	10	4	
Biliary	8	2	0.520
Nonbiliary	2	2	
Trauma	7	5	
Location			> 0.99
Proximal (head or neck)	12	6	
Distal (body or tail)	5	3	
PFCs	7	6	0.411
Diameter of PFC (cm)^a^	2.5 (2.0~7.6)	5.1 (2.0~11.4)	0.719
Interval between discovering pseudocyst or fistula and DPDS (months)^a^	7.0 (0.5~84)	4.0 (1.0~64)	0.686
ETD	13 (76.5%)	2 (22.2%)	0.014
Stent retention (days)^a^	137 (95–180)	90 (15–356)	0.396
≥2 stent insertion	5 (29.4%)	1 (11.1%)	0.604
Pancreatic debridement before ERCP	4 (23.5%)	4 (44.4%)	0.382
CT guided puncture Before ERCP	8 (47.1%)	1 (11.1%)	0.098
EUS guided puncture Before ERCP	0 (0.0%)	2 (22.2%)	0.111
Developed DM	2 (11.8%)	1 (11.1%)	> 0.99
Developed diarrhea	3 (17.6%)	0 (0.0%)	0.529
Developed atrophy	8 (47.1%)	2 (22.2%)	0.399

^a^Described as median with range

*PFCs* peripancreatic fluid collections, *ETD* Endoscopic transpapillary drainage, *DM* diabetes mellitus

### Follow-up

The median follow-up time after diagnosis of DPDS was 40 months (range 22–110 months). Five of 31 patients (16.1%) were lost to follow-up after the last treatment including 1 patient with ANP and 4 with trauma (these 5 lived in provinces far from Shanghai, which might be a contributing factor). Two of them underwent ETD. During follow-up, among the patients who underwent ETD, 7 underwent one-time stent insertion, 5 underwent one-time stent exchange, and 1 had stent placement repeated 5 times. Thus, totally 24 stent placements were performed with 9 stents removed as planned and the remaining showed migration based on imaging studies. The median duration of stent therapy was 95 days (range 15–356 days). Four patients without ETD also showed a successful outcome. One patient with EPF underwent only a sphincterotomy, and the other 3 with EPFs showed clinical success attributable to pancreatic atrophy [1] or secondary to the maintenance of an internal sinus between the gastrointestinal tract and the pancreas, which was created using CT-guided percutaneous puncture tubes [2]. Nine patients (34.6%) did not improve clinically (Fig. 1). One patient underwent transmural drainage for sepsis after stent insertion. Eight patients needed surgery because no reduction was observed in the size of the PFC [5] and persistent external leakage [3]. Of the 8 patients, 1 underwent ETD, 2 underwent only sphincterotomy, and 5 did not undergo any transpapillary therapy. All 8 patients underwent pancreatojejunostomy during follow-up. During follow-up, cross-sectional imaging confirmed pancreatic atrophy in 10 patients (38.5%). Patients with disruption in the pancreatic head or neck (55.6%) were more likely to develop atrophy than those with body or tail disruption (0%) (*P* = 0.009) (Table 3). Three patients (11.5%) developed diabetes mellitus and 2 of them required insulin and 1 required metformin. Three (15.4%) of these patients developed exocrine pancreatic insufficiency; however, they were all treated with pancreatic enzymes.

**Table 3:** DPDS characteristics according to location of disruption

Characterristic	Proximal (head or neck) *N* = 22	Distal (body or tail) *N* = 9	*p* value
Age (year)^a^	36 (23~70)	30 (24~54)	0.877
Male	19 (86.4%)	5 (55.6%)	0.150
Etiology			0.704
AP	10	5	0.600
Biliary	6	4	
Nonbiliary	4	1	
Trauma	12	4	
Interval between discovering pseudocyst or fistula and DPDS (months)^a^	6.8 (0.5~84)	5.0 (1.0~48)	0.711
PFCs	11	5	> 0.99
Diameter of PFC (cm)^a^	2.5 (2.0~11.4)	5.6 (2.2~7.6)	0.571
ETD	11	6	0.456
≥ 2 stent insertion	3	3	0.622
Pancreatic debridement before ERCP	8 (36.4%)	1 (11.1%)	0.220
CT guided puncture Before ERCP	7 (31.8%)	4 (44.4%)	0.683
EUS guided puncture Before ERCP	1 (4.5%)	1 (11.1%)	0.503
Developed DM	2/18 (11.1%)	1/8 (11.1%)	> 0.99
Developed diarrhea	3/18 (16.7%)	0/8 (0.0%)	0.529
Developed atrophy	10/18 (55.6%)	0/8 (0.0%)	0.009

^a^Described as median with range

*PFCs* peripancreatic fluid collections, *ETD* Endoscopic transpapillary drainage, *DM* diabetes mellitus

## Discussion

**Fig. 3:**
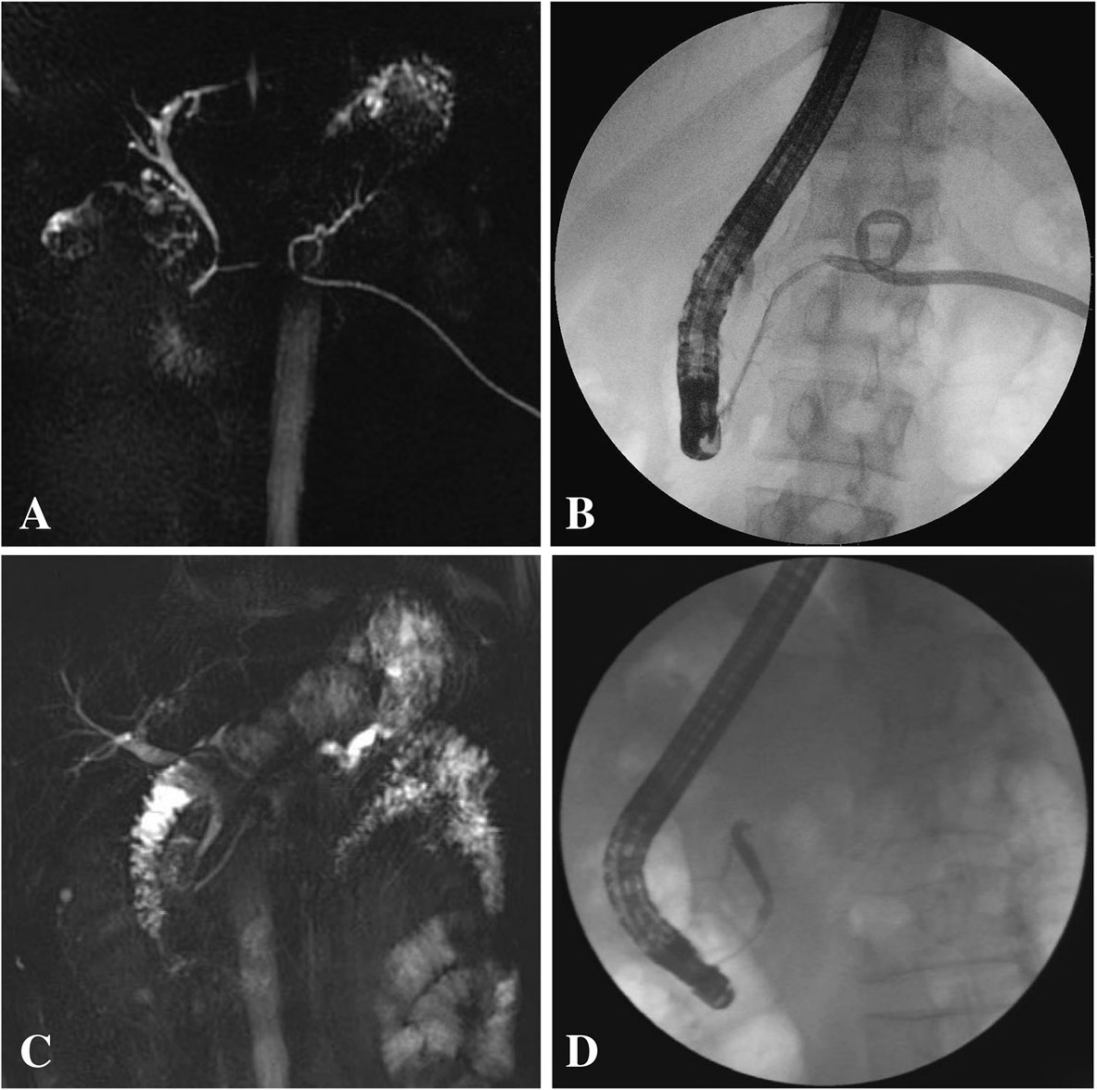
Two patients suspected of DPDS by MRCP before ERCP. Showing as **a**, **b** and **c**, **d** respectively. **a**, MRCP shows dilated main pancreatic duct in the tail with a fistula; **b**, Retrograde pancreatogram shows branch pancreatic duct and extravasation of contrast into drainage tube, without visualization of the pancreatic tail. C, MRCP shows dilated MPD in the tail; D, ERCP image shows complete cutoff of the proximal MPD

An accurate preoperative diagnosis of DPDS requires both, cross-sectional imaging (CT or MRI) and pancreatography. Although secretin-enhanced MRCP is proposed as an alternative to ERCP to diagnose DPDS, its sensitivity in demonstrating the site of the ductal disconnection is lower than that of ERCP [17]. ERCP is the gold standard to identify ductal disruption, which can be further defined as partial (opacification of the PD upstream to the site of disruption) or complete (nonopacification of the PD upstream to the leak) [12, 18–20]. Unlike MRCP, ERCP also serves as an endotherapeutic modality in selected patients with pancreatic injury. In this study, all patients routinely underwent MRCP or CT before ERCP and were diagnosed with DPDS using ERCP. An MRCP suggested DPDS in only 2 of 31 patients (Fig. 3). The median interval of DPDS in our study was 6.6 months, and much of this delay is attributable to a lack of awareness regarding the disease [21–23].

In our study, the ANP group showed a higher rate of CT-guided percutaneous drainage than the trauma group (*P* = 0.006). Pancreatic necrosis-induced infection and ductal leakage-induced PFCs usually necessitate percutaneous drainage. In many of these cases, DPDS was overlooked initially, and the patients were only diagnosed with DPDS once they develop an EPF after percutaneous drainage. In this condition, percutaneous drainage alone should be avoided for patients with DPDS because of the inevitable development of an EPF in these patients. Dual modality drainage is a good treatment option for these patients if the cyst is properly located. It involves the placement of percutaneous drains followed immediately by endoscopically placed transmural stents into the PFC. After transmural drainage is completed, the percutaneous drain is also opened. Once the fluid collection resolves, the percutaneous drain is removed [24]. We also observed that PD disruption was significantly associated with percutaneous drain placement [25, 26]. However, a causal relationship between them remains unclear. Gallstones were the most common cause of ANP (10/15, 67%), although any cause of pancreatitis can produce ductal leakage. Additionally, in concurrence with previously published articles [5, 25], DPDS occurred predominantly in the head or neck of pancreas (22/31, 71%) owing to the unique pancreatic anatomy that increases the susceptibility of this region. This group showed a higher tendency to develop pancreatic atrophy than that in the group with distal PD disconnections (*P* = 0.009). This is perhaps because pathology of head/neck duct affects the pancreatic parenchyma to a greater extent than disruptions in the body-tail area, where a small amount of viable pancreatic parenchyma exists upstream to the ductal disruption. Two of the 10 patients with pancreatic atrophy underwent pancreatojejunostomy during follow-up. The two patients had neither ETD nor sphincterotomy. Both of them had no recurrence of pancreatic fistula or pseudocyst after operation, but one patient still has abdominal pain.

Conventional treatment such as percutaneous drainage and medical treatments show a low success rate and high morbidity; thus, these are no longer recommended for DPDS. Surgical treatment including resection and derived procedures is accepted as a standard modality, and most physicians recommend derivd techniques, which were used in 8 patients as the first-line surgical treatment for DPDS [10, 11, 22, 23, 27, 28]. However, this approach demonstrates a higher morbidity and mortality than a nonsurgical approach [8–11, 29]. We attempted to identify predictors of successful outcomes following ETD to treat MPD disruption and observed that the best results were obtained in patients with partial PD disruption that can be bridged. However, in this study, 7 patients with DPDS could not undergo bridge treatment using stents but showed resolution of PFCs and clinical symptoms [12]. If these 7 patients can be considered clinically successful cases, the success rate of ETD in treating 23 patients with DPDS increases from 26 to 56.5%. It should be mentioned that it was impossibility to make bridging therapy in all cases in this study with the experienced doctors. Up to date, there was only a successful bridging in our center, which was reported in 2018 [30].

ETD was associated with clinical success in 87% (13/15) of patients with DPDS. This route of drainage is physiological because it uses the normal anatomical route of drainage of pancreatic secretions and does not involve the creation of an alternative route of drainage as is observed with transmural drainage. Thus, we attempted ETD before surgery, although 8 patients showed clinical failure and underwent surgery with complete cure without adverse events or recurrence during follow-up. ERCP can be associated with serious adverse events such as post-procedure pancreatitis and the risk of infection in sterile PFCs [31–33]. This study shows that ETD is safe and effective; however, postoperative infection was reported in 1 patient with PFC, which could be initially treated with transmural drainage. The diameter of the MPD limits the number and the size of the endoprosthesis that can be placed for drainage. The optimal duration of stent therapy remains unclear [12, 18]. In this study, stents remained in place over 3 months and no adverse events such as stent occlusion or stent-induced ductal changes were observed.

To our knowledge, the number of patients who met the diagnostic criteria of DPDS and were treated with transpapillary drainage was less than 31 [1, 10, 12, 13, 21, 34]. Limitations of our study: 1) this was a retrospectively designed study, 2) a small number of cases were included. Because of the low incidence of DPDS, it is difficult to study large patient cohorts. Nevertheless, our sample size is larger than those in previous reports, and notably, the follow-up duration is long enough, 3) a potential selection bias toward endoscopic transpapillary management vs. transmural or surgical management cannot be ignored, which is attributable in part, to our large and experienced ERCP practice and, 4) other drawbacks include that 16.1% of the patients were lost to follow-up. Evidence regarding DPDS is limited and this group of patients included in our study might represent a selected patient population with DPDS who underwent conventional multidisciplinary treatment.

## Conclusions

We present our experience with DPDS and emphasize that endoscopic management for DPDS is safe and is associated with favorable long-term outcomes. Additionally, the incidence of adverse events is low, and the failure rate was 13.3% (2/15). Thus, primary surgery is not required for all patients with DPDS. Systematic reviews and prospective, randomized multicenter trials are warranted in the future to validate the data regarding DPDS.

## Abbreviations

ANP: Acute necrotizing pancreatitis
CT: Contrast-enhanced computed tomography
DPDS: Disconnected pancreatic duct syndrome
EPF: External pancreatic fistula
ERCP: Endoscopic retrograde cholangiopancreatography
ETD: Endoscopic transpapillary drainage
MPD: Main pancreatic duct
MRCP: Magnetic resonance cholangiopancreatography
PD: Pancreatic ductal
PFC: Peripancreatic fluid collections
